# Author's reply

**Published:** 2010

**Authors:** Tarannum Fatima, Abadan K Amitava, Saba Siddiqui, Mohammad Ashraf

**Affiliations:** Institute of Ophthalmology, JN Medical College, Aligarh Muslim University, Aligarh - 202 001, India

Dear Editor,

We are thankful to Dr. Gopal,[1] for keenly perusing our article.[2] We agree that there exists evidence that suggests that recovery of binocularity is a likely outcome after successful squint surgery.[3] Our study only serves to substantiate that.

Although not stated separately, a simple arithmetic subtraction of the period of duration of strabismus from the age at presentation would have yielded the age of onset of strabismus. Remarkably the average was 6.6 ± 10 years, and with a median of one year.

We confess that the word ‘adult’, in the strictest sense does not deserve to be part of the title. Although not deliberate, we have used it in the sense of ‘visually mature’ patients being enrolled in this study. Even though it escaped the review process, the fault if any is entirely ours. Interestingly, there exist other articles using the word ‘adult’ which have included patients with a minimum age of 15 years![4,5]

Notably, 12 of the 15 patients had strabismus onset at ≤6 years; and eight at one year of age. This only justifies questioning the assumption whether disruption in the ‘critical period’ really is all that critical, since many of these patients, with early onset strabismus, gave favourable responses.

We agree that our study has largely exotropes and thus is more representative of them, and we have clearly said so. Still, one study in adults shows that 74.2% (23 of 31) of esotropes achieved postoperative fusion compared to 65% (52 of 80) of exotropes.[5] In fact, Kushner's research showed that 81% (60 of 74) of congenital esotropes demonstrated binocularity on Bagolini striated glasses (BSG).[3] Significantly, he reveals that in patients with best corrected visual acuity ≤ 20/200 in the deviated eye, 68% (45 of 66) demonstrate binocularity on BSG. Once again our preexisting notions, such as congenital esotropes or those with dense amblyopia being less likely to recover binocularity, need to be questioned.

Patients of cataracts with exotropia who recover binocularity postoperatively have little in common with our study.

Our basic message that visually mature patients, the majority with onset of strabismus in the ‘critical period’, are likely to recover binocularity following surgical realignment, still holds.
